# Regulation of Retroviral and SARS-CoV-2 Protease Dimerization and Activity through Reversible Oxidation

**DOI:** 10.3390/antiox11102054

**Published:** 2022-10-18

**Authors:** David A. Davis, Haydar Bulut, Prabha Shrestha, Hiroaki Mitsuya, Robert Yarchoan

**Affiliations:** HIV and AIDS Malignancy Branch, Center for Cancer Research, National Cancer Institute, Bethesda, MD 20814, USA

**Keywords:** human immunodeficiency virus, glutathionylation, dimerization, reversible oxidation, SARS-CoV-2 main protease, coronavirus, retrovirus, glutaredoxin, aspartyl protease, thioltransferase, methionine sulfoxide reductase

## Abstract

Most viruses encode their own proteases to carry out viral maturation and these often require dimerization for activity. Studies on human immunodeficiency virus type 1 (HIV-1), type 2 (HIV-2) and human T-cell leukemia virus (HTLV-1) proteases have shown that the activity of these proteases can be reversibly regulated by cysteine (Cys) glutathionylation and/or methionine oxidation (for HIV-2). These modifications lead to inhibition of protease dimerization and therefore loss of activity. These changes are reversible with the cellular enzymes, glutaredoxin or methionine sulfoxide reductase. Perhaps more importantly, as a result, the maturation of retroviral particles can also be regulated through reversible oxidation and this has been demonstrated for HIV-1, HIV-2, Mason-Pfizer monkey virus (M-PMV) and murine leukemia virus (MLV). More recently, our group has learned that SARS-CoV-2 main protease (M^pro^) dimerization and activity can also be regulated through reversible glutathionylation of Cys300. Overall, these studies reveal a conserved way for viruses to regulate viral polyprotein processing particularly during oxidative stress and reveal novel targets for the development of inhibitors of dimerization and activity of these important viral enzyme targets.

## 1. Introduction

A significant body of research on reversible *S*-glutathionylation of proteins (the formation of a reversible disulfide bond with cysteine (Cys)) has demonstrated that this process naturally occurs within cells and can regulate the function of many important target proteins [1,2,3]. Nonetheless, there is still debate as to the extent in which this process represents a significant mechanism to regulate normal biological processes as opposed to processes that function more under “abnormal” or disease-like conditions that result in oxidative stress. Infection with many pathogens can lead to oxidative stress, thereby decreasing the ratio of reduced glutathione (GSH) to oxidized glutathione (GSSG) in the cells and resulting in glutathionylation of susceptible protein thiols. Examples include the bacterial induced oxidative burst [4], ischemia [5,6,7,8] as well as oxidative stress resulting from viral infections [9,10,11,12,13,14,15]. For more information on this area the authors suggest a recent review on the role of glutathionylation in infection and inflammation by Checconi et al. [16]. Our interest has revolved around the effects of glutathionylation of viral proteases. The purpose of this article is to review some of the key points concerning the reversible oxidation/glutathionylation of viral proteases and how it may impact viral maturation and replication. We also discuss how these kinds of *in vitro* studies on reversible oxidation can reveal susceptible residues within known pathogen protein targets allowing for the development of new therapies. A variety of approaches can be taken to determine if *S*-glutathionylation is more or less likely to take place for a particular protein and if so whether the activity or function of the protein may be affected by the modification. While the only strict requirement for glutathionylation is the presence of a reduced form of Cys, there is substantial evidence that the location and the pKa of the -SH moiety (more reactive at lower pKa’s) can increase or decrease the likelihood of the modification. In one approach, if the protein of interest is purified it can be examined for the presence of Cys residues with that are not involved in disulfide bonds. Additionally, the conserved nature of these Cys residues (for example among related species) may point to an important role for that cysteine in a particular protein and the extent of surface exposure of the cysteine in the protein can be evaluated if the crystal structure of the protein is known. This limited type of analysis can, at least, inform potential leads for Cys residues that may undergo reversible glutathionylation/oxidation and affect or act to protect protein activity.

## 2. Regulation of Human Immunodeficiency Virus Type 1 (HIV-1) Protease through Reversible Glutathionylation

In 1991, Karlstrom and Levine demonstrated that purified HIV-1 protease, an aspartyl protease, was inactivated by copper and mercury and that this was dependent on the two conserved cysteines in the protease [17]. Subsequently, both cysteine 67 (Cys67) and cysteine 95 (Cys95) were found to have relatively low pKa’s (pKa about 6) in the native enzyme as compared to the typical pKa of about 8.5 for cysteines in unfolded proteins [18,19]. At the time, the goal was to identify novel targets (aside from the active site) for the development of HIV-1 protease inhibitors as the need for therapies for AIDS was at its peak. The initial studies primarily focused on targeting Cys67 as the crystal structure of HIV-1 protease solved by Wlodawer et al. demonstrated that Cys67 was a surface accessible residue while Cys95 was buried in the dimer interface [20]. The exposed nature of Cys67, it followed, provided an attractive target for inhibitors. In addition to the surface exposed nature of Cys67, it is directly adjacent to the sequence GlyHisLys. This tripeptide sequence forms a high-affinity complex with copper II and is a well-known growth factor present in human plasma [21]. Thus, these studies revealed that HIV-1 protease has two non-active site Cys residues in their reduced state with higher reactivity in the native protein (lower pKa’s) than typical Cys residues. It also revealed a potential exposed metal binding site (CysGlyHisLys) for the possible development of site-specific metal catalyzed allosteric inhibitors of HIV-1 protease. Although the GlyHisLys sequence is within the HIV-1 protease sequence, the region is surface exposed and may explain the reactivity of Cys67 to copper.

However, mutation of these Cys residues did not affect HIV-1 protease activity and so it was unclear why the virus would have evolved to have two conserved Cys residues within the protease that were otherwise unnecessary for protease activity [17]. One possibility was that perhaps the virus regulates protease activity through reversible modification of these residues to optimize viral replication. Due to the lowered pKa seen for the Cys67 and Cys95 along with viral infections generally leading to oxidative stress [13,14,22,23], S-glutathionylation taking place at these residues was plausible. In 1996, studies were carried out to assess if glutathionylation affected HIV-1 protease function and activity. Surprisingly, while previous studies suggested that covalent modification of Cys67 with Ellman’s reagent decreased protease activity [19], Davis et al. found that glutathionylation at Cys67 (using GSSG as the modifier of a C95A mutant protease) increased the catalytic activity 2-fold and also stabilized the enzyme from autoproteolysis (HIV-1 protease has three sites within its sequence that can be self-processed by HIV-1 protease) [24]. In stark contrast to the effects of glutathionylation of Cys67 was the finding that glutathionylation of Cys95 resulted in complete inhibition of protease activity [24]. In both cases, the effects were reversible with the addition of dithiothreitol (DTT), which removed the glutathione modifications and restored normal activity. It was proposed that glutathionylation of Cys95 might lead to disrupting dimerization of the protease thereby inhibiting protease activity. Inhibition of dimerization by glutathionylation was later demonstrated by analytical ultracentrifugation (AUC) [25] as well as size exclusion chromatography (SEC) [26] using an autoproteolysis resistant form of HIV-1 protease. While the buried nature of Cys95 in the mature protease dimer might make it a poor target for inhibitors, it remained possible that Cys95 may be exposed prior to excision from the poly-protein prior to viral maturation and budding. Additionally, this new information on the effects of glutathionylation on HIV-1 protease activity provided a possible explanation for the conserved nature of Cys67 as it could accelerate polyprotein processing under conditions favorable for glutathionylation particularly if Cys95 was inaccessible to modification within the dimer. Although Cys95 is buried within the mature dimer interface, inhibition of protease activity or activation via glutathionylation at Cys95 within the unprocessed polyprotein, where Cys95 might be more accessible, remained a possibility. 

Since the available technology at the time was not advanced enough to confirm glutathionylation of HIV-1 protease taking place in infected cells, studies focused on examining HIV-1 viral particles produced from mutant HIV-1 where the Cys residues of HIV-1 protease encoded instead for alanine (Ala). Somewhat surprisingly, it was found that mutating each Cys to Ala in HIV-1 protease in the context of the whole virus had no noticeable detrimental effect on viral infection or replication. In fact, studies examining virus production by measuring p24 capsid release indicated that, if anything, replication improved if either or both Cys residues were mutated to Ala suggesting that in chronically infected immortalized cells within the laboratory the presence of the Cys residues led to slower polyprotein processing (unpublished data). This suggested that if glutathionylation of HIV-1 protease were to take place in HIV-1 infected cells, it was not essential for virus replication, at least not under typical laboratory conditions. Further, the results suggesting improved replication for virions lacking the Cys residues supports the hypothesis that the Cys residues, through reversible oxidation, might aid in attenuating replication to decrease viral toxicity in infected cells that can occur upon viral release (viral lysis). To determine if oxidation of the Cys residues within HIV-1 protease might occur within the virus at all, studies were carried out by isolating immature HIV-1 virions (with the use of a potent HIV-1 protease active site inhibitor) encoding wild type (WT) or Cys-mutant proteases that were released from cells chronically infected with these virions [27]. These studies revealed that Cys95, in particular, was oxidized within the immature virions, and this impaired viral maturation which could be reversed with the addition of reducing agent [27]. Supporting the idea that modification of Cys95 was the key factor leading to a slower maturation rate, it was found that simply adding other sulfhydryl oxidizing agents (diamide or H_2_O_2_) to WT or C67A immature virions led to complete inhibition of maturation [27]. These studies demonstrated that Cys95 played a pivotal role in regulating viral maturation of immature virions. 

In immature virions, the protease is primarily retained within the Gag-Pro-Pol polyprotein. Thus, modification of Cys residues of protease may take place prior to its excision from the Gag-Pro-Pol polyprotein. It was predicted that Cys95 is more exposed to solvent when it is part of the larger polyprotein than when it is a mature protease. This would explain why Cys95, which is inaccessible within the dimer interface of the mature protease, might be modified prior to dimerization and therefore affect virion maturation. Indeed, using the “one-cut” assay it was found that adding the sulfhydryl oxidizing agent, diamide, blocked the initial step in WT Gag-Pro-Pol processing but had little effect on the Cys double mutant embedded protease construct [28]. These studies provided further data to implicate reversible oxidation of Cys residues in the protease in regulating polyprotein processing of the GagProPol polyprotein prior to the formation of the mature dimer.

**Figure 1:**
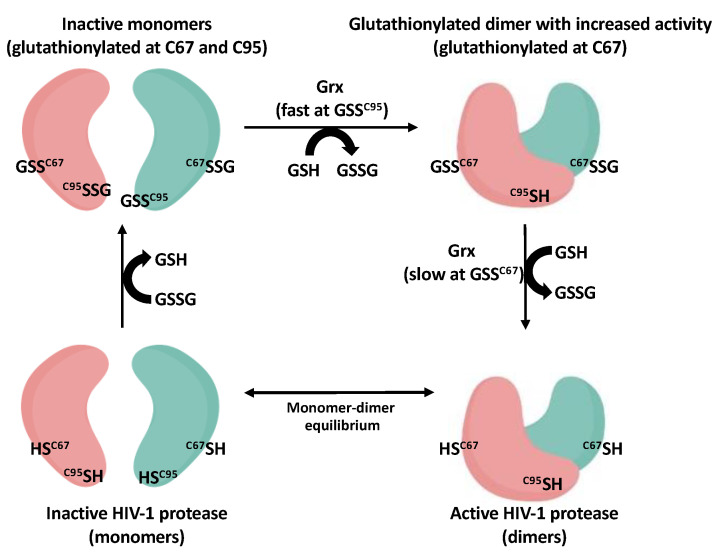
Model depicting HIV-1 protease monomers (**bottom left**) in equilibrium with its active dimer (**bottom right**). GSSG can glutathionylated Cys67 and Cys95 leading to inhibition of dimerization and activity (**top left**). Grx can rapidly remove glutathione from the Cys95 which results in dimerization and increased activity of the protease (**top right**). Grx more slowly removes glutathione from Cys67 producing the unmodified active dimer (**bottom right**).

Assuming that modifications of Cys95 was occurring through the common abundant low molecular weight thiol, glutathione, it reasoned that the process would likely be regulated by cellular enzymes. In collaboration with Mieyal and colleagues, our group demonstrated that low nanomolar concentrations of glutaredoxin (Grx) (also known as thioltransferase) could readily remove glutathione from Cys95 of HIV-1 protease resulting in protease specific activity that was greater than that seen for unmodified protease. Grx was much slower at removing glutathione from Cys67 but could ultimately restore the wild type unmodified enzyme and activity [29]. These data supported the likelihood that if the Cys residues did become modified by glutathione within cells under oxidative stress, that the net result might be an accumulation of a more active form of HIV-1 protease glutathionylated predominantly at Cys67 (because of the slower off rate at Cys67). Further supporting a role for Grx was the discovery of the presence of Grx within HIV-1 viral particles isolated from the media of infected cells [29]. Our current model for the regulation of HIV-1 protease through reversible glutathionylation is shown in Figure 1. As shown in Figure 1, the HIV-1 protease dimer is in equilibrium with inactive monomers. Cys67 and Cys95 of the protease can become glutathionylated in the presence of GSSG which prevents dimerization and activity. Grx can rapidly remove glutathione from Cys95 resulting in restoring dimerization and generating a form of HIV-1 protease with increased specific activity over that seen for wild type enzyme. Finally, Grx at a slower rate can remove glutathione from Cys67 and restore mature unmodified active HIV-1 protease dimer (Figure 1). These data provide a framework by which regulation of retroviral protease activity by reversible oxidation may take place in virus-infected cells. However, it should be made clear that while these studies support the possibility of reversible glutathionylation/oxidation of HIV-1 protease within cells they do not provide proof that glutathionylation of the protease is, in fact, taking place in infected cells and further studies are needed to assess this. However, as you will see in part 3 on the regulation of other retroviral proteases, there is accumulating evidence that oxidation of viral proteases is taking place in immature viral particles and that this process is reversible and affects viral maturation.

## 3. Regulation of Other Retroviral Proteases through Reversible Oxidation

As more and more sequences of HIV-1 isolates derived from patients were deposited it became clearer that Cys95 is much more highly conserved among isolates than Cys67. Additionally, under long-term HIV-1 protease inhibitor drug pressure, mutation of Cys95 to Phe95 was found to occur although only following the mutation of 10 other residues of the protease [26], providing evidence that while Cys95 is not required for HIV-1 infection and replication it nonetheless is relatively resistant to mutation. A potential argument against the concept that Cys95 might play a role in regulating HIV-1 protease activity was that HIV-2 protease (also a 99 amino acid aspartyl protease with high sequence similarity to HIV-1 protease) has no Cys residues at all. However, HIV-2 protease, has a conserved methionine (Met) at position 95 among the HIV-2 isolates and this is located at the identical region of the dimer interface as Cys95 of HIV-1 protease. Our group hypothesized that this may play a similar role in regulating HIV-2 protease activity and subsequently demonstrated that specific oxidation of Met95 with H_2_O_2_ led to inactivation of HIV-2 protease [30]. We further showed that this inactivation by H_2_O_2_ could be partially reversed in the presence of the cellular enzyme methionine sulfoxide reductase A (MsrA) [30]. The partial restoration of activity by MsrA was expected since it can only reverse the S diastereomer of methionine sulfoxide and H_2_O_2_ would generate both forms on the protease Met residues. MsrB was subsequently discovered and shown to reverse the R diastereomer [31]. It is now clear that cells contain Msr’s that can reverse both R and S forms of methionine sulfoxide [32,33]. Interestingly, the maturation of immature HIV-2 virions could be inhibited by the addition of H_2_O_2_ similar to that seen for HIV-1 virions supporting a role in regulating viral maturation [30]. Similar experiments were also carried out with HIV-1 protease whose Cys95 was mutated to Met. This enzyme could be oxidized at Met95 to the sulfoxide and activity completely inhibited. The inhibition was reversible with methionine sulfoxide reductase [30]. These studies revealed the possibility of an alternate mechanism of reversible oxidation in regulating protease activity through reversible oxidation of Met residues. These findings also demonstrated the sensitive nature of the beta sheets making up the dimer interface of HIV protease as the simple oxidation of a Met residue in each monomer can reversibly inhibit dimerization and activity.

Additional insights into the regulation of retrovirus proteases came from elegant studies carried out on Mason-Pfizer monkey virus (M-PMV) by Parker and Hunter [34]. Unlike most retroviruses, in which the virus particles mature on the cell surface, M-PMV polyproteins assemble in the cytoplasm. However, these particles remain immature in the cytoplasm. Parker and Hunter showed that immature M-PMV particles could be activated to processing by the addition of reducing agent [34] similar to what is observed with immature HIV-1 virions [27]. Subsequent studies on M-PMV revealed that an unusual intramolecular Cys disulfide can form within the protease and increase protease activity [35]. This work provides evidence suggesting that inactivation of M-PMV Gag by exposure to oxidizing conditions in the cytoplasm plays an important role in preventing premature maturation and release of active protease (which could be toxic to the host cell) before viral budding. Similarly, it was demonstrated that addition of reducing agent to murine leukemia virus (MLV) immature particles produced in the presence of a mild oxidizing agent, disulfide-substituted benzamide-2, induced their maturation *in vitro* [36]. While the MLV protease does not have a Cys or Met residue predicted at its dimer interface, it does contain a single highly conserved Cys residue [25]. It is reasonable to speculate that this protease is also regulated through reversible oxidation at this Cys residue, although its effect on protease activity may be through an alternate mechanism rather than through blocking dimerization. Nevertheless, the accumulating evidence points to reversible oxidation of retroviral protease activity as playing a distinct role in regulating the activation of polyprotein processing in retroviruses. Glutathionylation of other viral proteins besides the proteases may also take place. For example, it was recently demonstrated that glutathionylation affects guanylyl transferase and RNA-dependent RNA polymerase activities of Zika Ns5 proteins [37].

**Figure 2:**
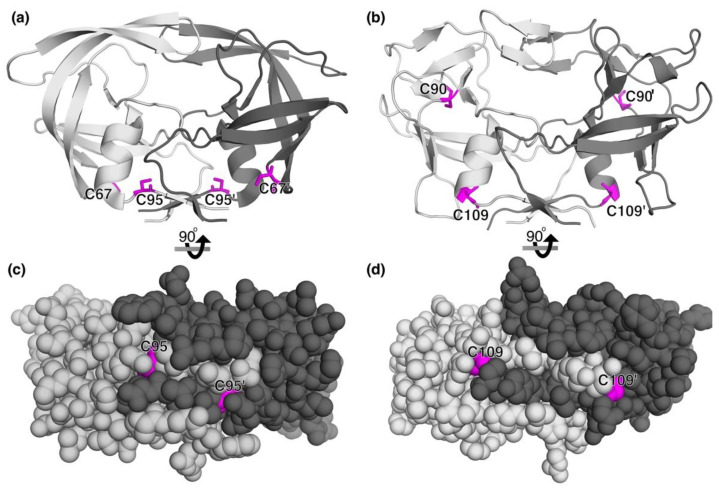
Side-by-side comparison of the ribbon and space filling models for HIV-1 (**a**,**c**) (PDB:4LL3) and HTLV-1 (**b**,**d**)) (PDB:4YDF) proteases. The location of the two Cys residues in each protease is depicted in magenta in the ribbon drawings (**top**). The space filling models below each ribbon model are rotated 90 degrees to show the location of Cys95 (for HIV-1) and Cys109 (for HTLV-1) within the beta sheet interface regions (in magenta, **bottom**).

The revelations regarding reversible oxidation of HIV-1, HIV-2, and M-PMV led us to explore if other, if not most, retroviral proteases might also be regulated through reversible oxidation (glutathionylation, methionine oxidation, disulfide formation, nitrosylation, etc.) taking place at the dimer interface regions of these dimeric enzymes. Our group explored this possibility by first examining sequence alignments for the predicted interface regions of several different retroviral proteases and found that most of the retroviral proteases had Met or Cys, or both, near or within their predicted dimer interface regions [25]. For example, human T-cell leukemia virus type 1 (HTLV-1) protease has two Cys residues at position 90 and 109. Cys109 was predicted by sequence alignment to be adjacent to the dimer interface region although no structure was available at the time of publication of the alignments. Our group assessed if glutathionylation of the HTLV-1 protease might regulate HTLV-1 protease activity. Indeed, studies revealed that HTLV-1 protease could be regulated by reversible glutathionylation much like HIV-1 protease [25]. The activity could also be restored by adding the ubiquitous cellular enzyme Grx which could remove glutathione from both Cys residues of HTLV-1. The crystal structure of HTLV-1 protease was eventually solved [38] and so an inspection of the location of the Cys residues could be done. A comparison of the dimeric crystal structure for HIV-1 protease and HTLV-1 protease and the location of the Cys residues within the interface regions is shown in Figure 2. The crystal structure for HIV-1 (Figure 2a,c) shows the intimate association of Cys95 within the dimer interface and the crystal structure of HTLV-1 protease (Figure 2b,d) shows a similar involvement of Cys109 present at the end of the helix that positions the interface peptides for dimerization (Figure 2b). Thus, oxidation or glutathionylation of Cys95 in HIV-1 protease and Cys109 in HTLV-1 protease could sterically interfere with beta sheet formation and dimerization. In summary, there is data to demonstrate that the activity of several different retroviral proteases is regulated by reversible oxidation, and these include the proteases from HIV-1, HIV-2, M-PMV, HTLV-1, and MLV.

## 4. Regulation of SARS-CoV-2 Main Protease through Reversible Glutathionylation

In December of 2019, a widespread infection of lethal novel coronavirus, now called SARS-CoV-2 was observed in Wuhan, China. SARS-CoV-2 soon spread throughout the world and led to the coronavirus-19 (COVID-19) pandemic; it was contained only with dramatic public health measures including widespread lockdowns. Even with the rapid development of effective vaccines, as of the summer of 2022, SARS-CoV-2 has killed more than one million persons in the United States and over 6 million persons worldwide. Based on research done previously on the main protease (M^pro^) of SARS-CoV-1 and other coronaviruses, it was learned that this cysteine protease was essential for viral replication and provided a promising target for the development of inhibitors of viral replication [39]. This holds true for SARS-CoV-2 M^pro^ which is strikingly similar in sequence and structure between the two viruses (both proteases are cysteine proteases of 306 amino acids in length and 96% conservation). 

**Figure 3:**
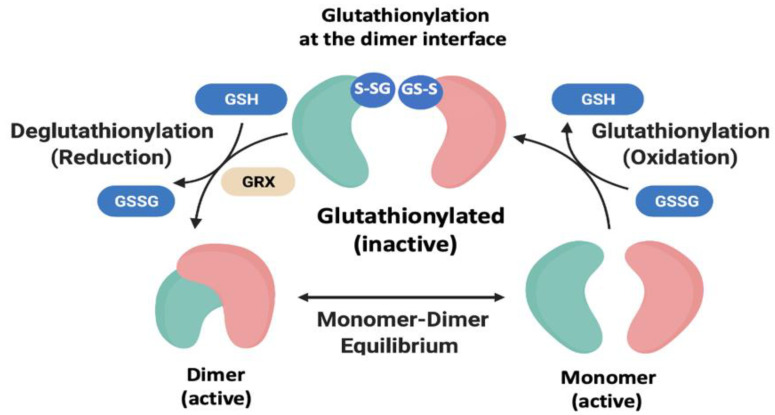
Model depicting the M^pro^ active dimer in equilibrium with its inactive monomers. Oxidized glutathione can glutathionylate Cys300 leading to inhibition of dimerization and activity. This process can be reversed with reducing agents or with Grx leading to the fully active dimer form.

M^pro^ of SARS-CoV-2 is encoded as part of two large polyproteins, pp1a and pp1ab; once released from the polyproteins by self-cleavage, M^pro^ is responsible for at least 10 additional cleavages during viral replication that in turn release additional M^pro^ and additional proteins needed for virus production. Dimerization of M^pro^ from SARS-CoV-2 is mediated by the C-terminal domain III a feature unique among the *Coronaviridae* family as this domain is absent in the *Picornaviridae* and *Caliciviridae* families [40]. Thus, M^pro^ of SARS-CoV-2 requires dimerization to be enzymatically active [39,41]. Interestingly, M^pro^ contains 12 reduced Cys residues within each monomer one of which is the active site Cys residue 145. It has been suggested that the high number of cysteines in M^pro^ may function to protect the active site Cys from oxidative insult [42]. Our group explored the potential for the cysteines of M^pro^ to be modified by glutathionylation and as a result possibly affect the dimerization and/or activity of the enzyme. Using the expertise derived from studies of HIV-1 protease, we found that dimerization of SARS-CoV-2 main protease could be reversibly regulated through glutathionylation at Cys300; this Cys residue is at the dimer interface and not part of the active site. Moreover, the active site Cys145 was not found to be a significant target for glutathionylation [43]. Inhibition of dimerization by glutathionylation was confirmed by analytical ultracentrifugation as well as size-exclusion chromatography like that carried out for HIV-1 protease. Activity of the glutathionylated protease could be restored with reducing agents or in the presence of glutaredoxin. M^pro^ with a mutation of Cys300 to Ser300 was no longer inhibited by glutathionylation even though another Cys residue in C300S was still glutathionylated. Thus, although two of the 12 Cys residues appeared relatively reactive to GSSG at a pH of 7.0, only glutathionylation of Cys300 appeared to affect dimerization and activity. It was further found that the glutathionylation of Cys300 could be reversed with Grx. Our current model depicting the reversible glutathionylation for M^pro^ is shown in Figure 3. However, it remains possible that reversible oxidation of M^pro^ activity occurs in pp1a and/or pp1ab polyproteins, as in retroviral Gag-Pro-Pol, but this has not yet been experimentally shown. 

These studies mirrored the data obtained for HIV-1 protease and revealed that regulation of dimerization through reversible oxidation may extend beyond the retrovirus family. However, a noteworthy difference is that the M^pro^ of SARS-CoV-2 acts at a relatively early step in viral replication rather than just before viral budding as is the case with retroviral proteases. SARS-CoV-2 is thought to have jumped to humans from a closely related virus in bats. Bats sustain prolonged infection with many viruses without developing an inflammatory response, which could deplete the scant energy reserves of the bats. One can speculate that the inactivation of SARS-CoV-2 M^pro^ by glutathionylation may have evolved as a way of preventing the virus from killing off the host bats when they are under oxidative stress from expending excess metabolic energy, for example when migrating. Previous studies have demonstrated that during migration bats are placed under a high level of oxidative stress [44,45,46].

## 5. The Dimer Interface of Viral Proteases as A Novel Antiviral Target

While the basic biology surrounding glutathionylation of HIV-1 protease is still incomplete and requires further investigation, these studies reveal new ways to target the protease that does not rely on the active site. Additionally, combinations of an active site inhibitor and a dimerization inhibitor of HIV-1 protease very likely could improve inhibition of protease activity and replication and decrease the chances for the development of resistance mutations needed to outwit these inhibitors. As a proof of concept, Davis et al. developed cell permeable peptide dimerization inhibitors that mimicked the N and C-terminal regions of HIV-1 protease which make up the dimer interface [47]. Although these peptides inhibited protease activity by blocking dimerization of the purified protease, by themselves they showed little activity against the viral protease processing in HIV-1 infected cells. However, in combination with suboptimal concentrations of different active site inhibitors, the addition of the peptide resulted in greater inhibition of polyprotein processing as evidenced by increases in the p55 polyprotein and decreases in the p24 capsid protein [47]. These studies provide a proof of concept for the combined use of active site and dimerization inhibitors of HIV-1 protease in blocking HIV-1 replication. Cys95 is also an attractive target for Cys modification and inhibition of protease activity providing a new way to block protease activity as opposed to targeting the active site. Indeed, in 1994, De Voss et al. identified haloperidol-based irreversible inhibitors of HIV-1 and HIV-2 protease [48]. Their studies revealed that these inhibitors were 4–80 times more potent on HIV-1 protease than HIV-2 protease and this improved inhibition was ascribed to irreversible alkylation of the Cys residues of HIV-1 protease (HIV-2 has no Cys residues). A subsequent follow-up study revealed that the Cys residues were indeed targeted and modified, with Cys95 becoming completely derivatized by haloperidol while Cys67 was 75% modified [49]. Together these studies suggest that HIV-1 protease activity could also be inhibited (irreversible alkylation) by targeting Cys residues but particularly Cys95. Similarly, Cys300 of Sars-CoV-2 M^pro^ might also be targeted by Cys alkylation agents or other Cys modifying agents and block dimerization and activity. Recently, a hydrophobic pocket consisting of Ile21, Leu253, Gln256 and Cys300 of M^pro^ was identified as a binding site for two different compounds [50] and could be used as a starting point to target Cys300 and dimerization. Interestingly, a month after our work which revealed Cys300 as a potential target for glutathionylation and inhibition of activity, Xia and colleagues demonstrated that colloidal bismuth subcitrate as an allosteric inhibitor of SARS-CoV-2 M^pro^ and that it could inhibit SARS-CoV-2 replication [51]. In their report, Cys300 was required for the binding of one unit of bismuth to the main protease and, based on size exclusion chromatography, resulted in inhibition of dimerization. These data further demonstrate that Cys300 and the dimer interface region can be an additional target for the development of new therapies against SARS-CoV-2 replication. It is noteworthy that the production of infectious virions of HIV-1 and Sars-CoV-2 requires activation of an embedded protease within the viral polyproteins. Both viral proteases require dimerization for activity and therefore blocking dimerization at the early stages of replication or at later stages could both be a viable way to block virus production.

## 6. Conclusions

The cumulative data on oxidation of viral proteases shows that several viruses have evolved to have dimeric proteases whose activity can be modulated by reversible oxidation of susceptible residues (Cys or Met). The role that these reactive residues play in vivo remains uncertain but can include (1) regulating (inhibiting or enhancing) enzyme activity during oxidative stress (2) protecting the protein from oxidative damage and/or (3) acting as sensors to the environment so that replication only occurs under favorable conditions. Modification of residues at the interface of these enzymes can block dimerization, likely through steric hinderance, while modification at alternative sites may favor increased activity (for example Cys67 of HIV-1 protease) or act as decoys to prevent damaging oxidative events that could ultimately irreversibly damage the enzyme or the enzymes active site. The inhibition of the proteases by modification of susceptible residues appears to enable the viruses to optimize replication and perhaps prevent the premature killing of the host cell by the viral protease. In the case of retroviruses, inhibition of protease activity by modification of proteases at the dimer interface prevents the early cleavage of GagProPol polyprotein. In the case of SARS-CoV-2, we can speculate that reversible oxidation of the protease may serve to prevent the activity of protease and thus viral replication when the host cell is under oxidative stress, and this may have prevented the virus from killing the host bats when they are expending excess metabolic energy. Modification of other Cys residues of SARS-CoV-2 by reversible glutathionylation also may serve to protect the active site cysteine from modification. To this point, M^pro^ of SARS-CoV-2 has 12 reduced Cys residues within each monomer. Having Cys and/or Met amino acid residues that can shield oxidative insult following infection and at the same time be able to undergo reduction through cellular enzymes is an attractive way to protect important viral proteins. Still, it is not clear which reversible modifications (glutathionylation, nitrosylation, palmitoylation, sulfenic acid or sulfoxide formation, etc.) occur in these viral proteins in infected cells and the extent to which these modifications are affecting the rate of viral replication. Recent advances in protein sequencing using mass spectrometry as well as new ways to detect modifications such as anti-glutathione antibodies should pave the way to better understand these biological processes and their roles. Finally, regardless of how significant the role of reversible oxidation is biologically for these viral enzymes, these studies have revealed alternative ways to design inhibitors that can block the function of these important therapeutic targets for these deadly diseases.

## References

[1] Gallogly M.M., Starke D.W., Mieyal J.J. (2009). Mechanistic and kinetic details of catalysis of thiol-disulfide exchange by glutaredoxins and potential mechanisms of regulation. Antioxid. Redox Signal..

[2] Shelton M.D., Chock P.B., Mieyal J.J. (2005). Glutaredoxin: Role in reversible protein s-glutathionylation and regulation of redox signal transduction and protein translocation. Antioxid. Redox Signal..

[3] Shelton M.D., Mieyal J.J. (2008). Regulation by reversible S-glutathionylation: Molecular targets implicated in inflammatory diseases. Mol. Cells.

[4] Gorlach A., Dimova E.Y., Petry A., Martinez-Ruiz A., Hernansanz-Agustin P., Rolo A.P., Palmeira C.M., Kietzmann T. (2015). Reactive oxygen species, nutrition, hypoxia and diseases: Problems solved?. Redox Biol..

[5] Yapa Abeywardana M., Samarasinghe K.T.G., Munkanatta Godage D., Ahn Y.H. (2021). Identification and Quantification of Glutathionylated Cysteines under Ischemic Stress. J. Proteome Res..

[6] Burns M., Rizvi S.H.M., Tsukahara Y., Pimentel D.R., Luptak I., Hamburg N.M., Matsui R., Bachschmid M.M. (2020). Role of Glutaredoxin-1 and Glutathionylation in Cardiovascular Diseases. Int. J. Mol. Sci..

[7] Matsui R., Ferran B., Oh A., Croteau D., Shao D., Han J., Pimentel D.R., Bachschmid M.M. (2020). Redox Regulation via Glutaredoxin-1 and Protein S-Glutathionylation. Antioxid. Redox Signal..

[8] Zweier J.L., Chen C.A., Druhan L.J. (2011). S-glutathionylation reshapes our understanding of endothelial nitric oxide synthase uncoupling and nitric oxide/reactive oxygen species-mediated signaling. Antioxid. Redox Signal..

[9] Popa G.L., Popa M.I. (2022). Oxidative Stress in Chronic Hepatitis B-An Update. Microorganisms.

[10] Galli F., Marcantonini G., Giustarini D., Albertini M.C., Migni A., Zatini L., Gioiello A., Rossi R., Bartolini D. (2022). How Aging and Oxidative Stress Influence the Cytopathic and Inflammatory Effects of SARS-CoV-2 Infection: The Role of Cellular Glutathione and Cysteine Metabolism. Antioxidants.

[11] Mathew S.S., Bryant P.W., Burch A.D. (2010). Accumulation of oxidized proteins in Herpesvirus infected cells. Free Radic. Biol. Med..

[12] Lassoued S., Ben Ameur R., Ayadi W., Gargouri B., Ben Mansour R., Attia H. (2008). Epstein-Barr virus induces an oxidative stress during the early stages of infection in B lymphocytes, epithelial, and lymphoblastoid cell lines. Mol. Cell. Biochem..

[13] Ciriolo M.R., Palamara A.T., Incerpi S., Lafavia E., Bue M.C., De Vito P., Garaci E., Rotilio G. (1997). Loss of GSH, oxidative stress, and decrease of intracellular pH as sequential steps in viral infection. J. Biol. Chem..

[14] Pace G.W., Leaf C.D. (1995). The role of oxidative stress in HIV disease. Free Radic. Biol. Med..

[15] Gonzalez-Dosal R., Horan K.A., Rahbek S.H., Ichijo H., Chen Z.J., Mieyal J.J., Hartmann R., Paludan S.R. (2011). HSV infection induces production of ROS, which potentiate signaling from pattern recognition receptors: Role for S-glutathionylation of TRAF3 and 6. PLoS Pathog..

[16] Checconi P., Limongi D., Baldelli S., Ciriolo M.R., Nencioni L., Palamara A.T. (2019). Role of Glutathionylation in Infection and Inflammation. Nutrients.

[17] Karlstrom A.R., Levine R.L. (1991). Copper inhibits the protease from human immunodeficiecy virus 1 by both cysteine-dependent and cysteine-independent mechanisms. Proc. Natl. Acad. Sci. USA.

[18] D’Ettorre C., Levine R.L. (1994). Reactivity of cysteine-67 of the human immunodeficiency virus-1 protease: Studies on a peptide spanning residues 59 to 75. Arch. Biochem. Biophys..

[19] Karlstrom A.R., Shames B.D., Levine R.L. (1993). Reactivity of cysteine residues in the protease from human immunodeficiency virus: Identification of a surface-exposed region which affects enzyme function. Arch. Biochem. Biophys..

[20] Wlodawer A., Miller M., Jaskolski M., Sathyanarayana B.K., Baldwin E., Weber I.T., Selk L.M., Clawson L., Schneider J., Kent S.B.H. (1989). Conserved folding in retroviral proteases: Crystal structure of a synthetic HIV-1 protease. Science.

[21] Pickart L., Lovejoy S. (1987). Biological activity of human plasma copper-binding growth factor glycyl-L-histidyl-L-lysine. Methods Enzym..

[22] Aukrust P., Svardal A.M., Muller F., Lunden B., Berge R.K., Ueland P.M., Froland S.S. (1995). Increased levels of oxidized glutathione in CD4+ lymphocytes associated with disturbed intracellular redox balance in human immunodeficiency virus type 1 infection. Blood.

[23] Schwarz K.B. (1996). Oxidative stress during viral infection: A review. Free Radic. Biol. Med..

[24] Davis D.A., Dorsey K., Wingfield P.T., Stahl S.J., Kaufman J., Fales H.M., Levine R.L. (1996). Regulation of HIV-1 protease activity through cysteine modification. Biochemistry.

[25] Davis D.A., Brown C.A., Newcomb F.M., Boja E.S., Fales H.M., Kaufman J., Stahl S.J., Wingfield P., Yarchoan R. (2003). Reversible oxidative modification as a mechanism for regulating retroviral protease dimerization and activation. J. Virol..

[26] Davis D.A., Tebbs I.R., Daniels S.I., Stahl S.J., Kaufman J.D., Wingfield P., Bowman M.J., Chmielewski J., Yarchoan R. (2009). Analysis and characterization of dimerization inhibition of a multi-drug-resistant human immunodeficiency virus type 1 protease using a novel size-exclusion chromatographic approach. Biochem. J..

[27] Davis D.A., Yusa K., Gillim L.A., Newcomb F.M., Mitsuya H., Yarchoan R. (1999). Conserved cysteines of the human immunodeficiency virus type 1 protease are involved in regulation of polyprotein processing and viral maturation of immature virions. J. Virol..

[28] Daniels S.I., Davis D.A., Soule E.E., Stahl S.J., Tebbs I.R., Wingfield P., Yarchoan R. (2010). The initial step in human immunodeficiency virus type 1 GagProPol processing can be regulated by reversible oxidation. PLoS ONE.

[29] Davis D.A., Newcomb F.M., Starke D.W., Ott D.E., Mieyal J.M., Yarchoan R. (1997). Thioltransferase (glutaredoxin) is detected within HIV-1 and can regulate the activity of glutathionylated HIV-1 protease in vitro. J. Biol. Chem..

[30] Davis D.A., Newcomb F.M., Moskovitz J., Wingfield P.T., Stahl S.J., Kaufman J., Fales H.M., Levine R.L., Yarchoan R. (2000). HIV-2 protease is inactivated after oxidation at the dimer interface and activity can be partly restored with methionine sulphoxide reductase. Biochem. J..

[31] Moskovitz J., Singh V.K., Requena J., Wilkinson B.J., Jayaswal R.K., Stadtman E.R. (2002). Purification and characterization of methionine sulfoxide reductases from mouse and Staphylococcus aureus and their substrate stereospecificity. Biochem. Biophys. Res. Commun..

[32] Kaya A., Lee B.C., Gladyshev V.N. (2015). Regulation of protein function by reversible methionine oxidation and the role of selenoprotein MsrB1. Antioxid. Redox Signal..

[33] Stadtman E.R., Moskovitz J., Berlett B.S., Levine R.L. (2002). Cyclic oxidation and reduction of protein methionine residues is an important antioxidant mechanism. Mol. Cell. Biochem..

[34] Parker S.D., Hunter E. (2001). Activation of the Mason-Pfizer monkey virus protease within immature capsids in vitro. Proc. Natl. Acad. Sci. USA.

[35] Zabranska H., Tuma R., Kluh I., Svatos A., Ruml T., Hrabal R., Pichova I. (2007). The role of the S-S bridge in retroviral protease function and virion maturation. J. Mol. Biol..

[36] Campbell S., Oshima M., Mirro J., Nagashima K., Rein A. (2002). Reversal by dithiothreitol treatment of the block in murine leukemia virus maturation induced by disulfide cross-linking. J. Virol..

[37] Saisawang C., Kuadkitkan A., Auewarakul P., Smith D.R., Ketterman A.J. (2018). Glutathionylation of dengue and Zika NS5 proteins affects guanylyltransferase and RNA dependent RNA polymerase activities. PLoS ONE.

[38] Li M., Laco G.S., Jaskolski M., Rozycki J., Alexandratos J., Wlodawer A., Gustchina A. (2005). Crystal structure of human T cell leukemia virus protease, a novel target for anticancer drug design. Proc. Natl. Acad. Sci. USA.

[39] Anand K., Ziebuhr J., Wadhwani P., Mesters J.R., Hilgenfeld R. (2003). Coronavirus main proteinase (3CLpro) structure: Basis for design of anti-SARS drugs. Science.

[40] Ho C.Y., Yu J.X., Wang Y.C., Lin Y.C., Chiu Y.F., Gao J.Y., Lai S.J., Chen M.J., Huang W.C., Tien N. (2022). A Structural Comparison of SARS-CoV-2 Main Protease and Animal Coronaviral Main Protease Reveals Species-Specific Ligand Binding and Dimerization Mechanism. Int. J. Mol. Sci..

[41] Xia B., Kang X. (2011). Activation and maturation of SARS-CoV main protease. Protein. Cell.

[42] Ravanfar R., Sheng Y., Shahgholi M., Lomenick B., Jones J., Chou T.F., Gray H.B., Winkler J.R. (2022). Surface cysteines could protect the SARS-CoV-2 main protease from oxidative damage. J. Inorg. Biochem..

[43] Davis D.A., Bulut H., Shrestha P., Yaparla A., Jaeger H.K., Hattori S.I., Wingfield P., Mitsuya H., Yarchoan R. (2021). Regulation of the Dimerization and Activity of SARS-CoV-2 Main Protease through Reversible Glutathionylation of Cysteine 300. bioRxiv.

[44] Chionh Y.T., Cui J., Koh J., Mendenhall I.H., Ng J.H.J., Low D., Itahana K., Irving A.T., Wang L.F. (2019). High basal heat-shock protein expression in bats confers resistance to cellular heat/oxidative stress. Cell Stress Chaperones.

[45] Costantini D., Lindecke O., Petersons G., Voigt C.C. (2019). Migratory flight imposes oxidative stress in bats. Curr. Zool..

[46] Wilhelm Filho D., Althoff S.L., Dafre A.L., Boveris A. (2007). Antioxidant defenses, longevity and ecophysiology of South American bats. Comp. Biochem. Physiol. C Toxicol. Pharm..

[47] Davis D.A., Brown C.A., Singer K.E., Wang V., Kaufman J., Stahl S.J., Wingfield P., Maeda K., Harada S., Yoshimura K. (2006). Inhibition of HIV-1 replication by a peptide dimerization inhibitor of HIV-1 protease. Antivir. Res..

[48] De Voss J.J., Sui Z., DeCamp D.L., Salto R., Babe L.M., Craik C.S., Ortiz de Montellano P.R. (1994). Haloperidol-based irreversible inhibitors of the HIV-1 and HIV-2 proteases. J. Med. Chem..

[49] Salto R., Babe L.M., Li J., Rose J.R., Yu Z., Burlingame A., De Voss J.J., Sui Z., Ortiz de Montellano P., Craik C.S. (1994). In vitro characterization of nonpeptide irreversible inhibitors of HIV proteases. J. Biol. Chem..

[50] Gunther S., Reinke P.Y.A., Fernandez-Garcia Y., Lieske J., Lane T.J., Ginn H.M., Koua F.H.M., Ehrt C., Ewert W., Oberthuer D. (2021). X-ray screening identifies active site and allosteric inhibitors of SARS-CoV-2 main protease. Science.

[51] Tao X., Zhang L., Du L., Liao R., Cai H., Lu K., Zhao Z., Xie Y., Wang P.H., Pan J.A. (2021). Allosteric inhibition of SARS-CoV-2 3CL protease by colloidal bismuth subcitrate. Chem. Sci..

